# Supplementary material for: Patterns of Intron Gain and Loss in Fungi
Source: PLoS Biol. 2004 Nov 30;2(12):e422. doi: 10.1371/journal.pbio.0020422 (PMC532390; doi:10.1371/journal.pbio.0020422)
Supplement: Table S1 — Also available at http://genes.mit.edu/NielsenEtAl/. (4.3 MB ZIP). [file pbio.0020422.st001.zip › NielsenEtAl/html/996.html]

AN5914.1.NCU03032.1.MG01330.1.FG00742.1


```
 CLUSTAL W (1.82) Multiple Sequence Alignments - Introns Inserted


Sequence 1: MG01330.1	832 aa
Sequence 2: FG00742.1	835 aa
Sequence 3: NCU03032.1	908 aa
Sequence 4: AN5914.1	790 aa
Alignment Length: 976 aa
Number Identitical Residues: 324 aa
Alignment Score (without introns) 17130


MG01330.1 	---------M~DSNK--------------------------LIFTGLGTLLC--LGIGLS
NCU03032.1	MGDNSAMASH~GGHMGNISLPSLSVTRTLADLNFNTTTTKSIFFTGVAVLVFLVTTSNYS
FG00742.1 	------MASH0GGNM--------------------------LSLANAQSLLAGIVAAALS
AN5914.1  	------MSSL~ASGPAGFLLSG--------------FDRHYASIAGAAFFVCALVAVVLV
          	       ::   .  ..   ..                     ::.   ::         

MG01330.1 	RLLNK--QGPKTGDQSPGLLQAFGLFFYTCFVKPHR-VGGTN~TQQDALESFYETQASIY
NCU03032.1	RKTTKN-EDDNEDEGNPSSLKSLLLFCYSCFIKPHATAGTTG~TQQDALESFYRSQADIY
FG00742.1 	TLCLTSVFSSKSQDESPGLIKSFFLFFYSSFLKPHQ-GDSKG~NQQDALESFYKKQAGAY
AN5914.1  	VASQKR----NKVDNNSG-ISVYLRFIYASFLKPHD----KG1CQLTAP------QATVY
          	    .     :  : ... :.    * *:.*:***     ..  *  *       **  *

MG01330.1 	DVTRTTLLKGREDMLALAAAQLLHRAKK------------------------------QS
NCU03032.1	DATRGTLLKGREDMLALAASQLRYKVEAGLGGLGGAGDGLEKRQRNGKTCVTVAGTGTGT
FG00742.1 	DATRKVLLRGREDMLALVAAQMQAKLKD------------------------------NK
AN5914.1  	DATRKRLLRGREDMLGLVAAQLKYKVENK----------------------------ELR
          	*.**  **:******.*.*:*:  : :                                 

MG01330.1 	HQKRIWVD0IGGGTG~--WNIEAMNRFVDVPEFFSSVYLVDFSPSLCEVARQRFQRLGWK
NCU03032.1	RRKPIWVD0VGGGTG~--WNIEAMAKFVNVSEFFKTVYLVDFSPSLCEVARKRFARLGWE
FG00742.1 	KTKRVWVD0IGGGTG~--FNIEAMGAFVDVPTFFSSVYLVDFSPSLCEVAKKRFDRLGWK
AN5914.1  	KGKAIWVD0IGGGTG2SRYNIEAMAEFVPVPEFFSHVYLVDLSPSLLEVARQRFERLGWK
          	: * :*** :***** : :*****  ** *. **. *****:**** ***::** ****:

MG01330.1 	NVRVVCQDARKFRI-EDHEASGP---------------KGWNQ---AIGADLLTMSYSLS
NCU03032.1	NVRVICTDARKFRL-EDYEDVDEGESGSGDSSPSLSGWWGETKPGRHAGAELITMSYSLS
FG00742.1 	NVKVVCEDARKFRL-EDYESGMPSKSIPPRSPALSYFDKPRPD---FGGADLITMSYSLS
AN5914.1  	NVTVVCQDARAFQLPEDHIDPLKSVG---------------------AGADLVTMSYSLS
          	** *:* *** *::.**:     . .                      **:*:*******

MG01330.1 	MI0PDFYSVIDSLSSLLAPGGIMGVVDFYA~QSKVDFSFRNHTAGLTGRHCGFILRSFWR
NCU03032.1	MM0PDYFSIIDSLESLLAPHGLIAVVDFYA~QSKVDFTFRNYTGGLMNRHVGYFARNFWR
FG00742.1 	MI0PDYYSVIDSVTSLLSPQGIMGVVDFYV~QNKVDFAFRNYTGGLVDRHVNFLSRSFWR
AN5914.1  	MI~P-------------------------V1QSIVDVSSRNYIGGVFNRHVNWLGRAFWR
          	*: *                         . *. **.: **: .*: .** .:: * ***

MG01330.1 	AWFDLDRVSLEPARRDYLEYRFGTVLGVNTRNRMLGNIPYYIWIGCSKTSSGQNGSTQSL
NCU03032.1	SWFDADRVSLEPARRDYLEYRFGTVLTVNARNNTLGAIPYYIWLGCLKKPFSTSSLPHEI
FG00742.1 	SWFDLDRVGLEPSRRDYLEYKFGTVLNVNTRNKGLGAIPYYIWLGCHKKPFSSSSLPHEI
AN5914.1  	AWFEADRVNLDAARRDYLEYRFGTVISASERNYLLGGIPYYIFIGRQKDIYP-NQASREA
          	:**: ***.*:.:*******:****: .. **  ** *****::*  *     .  .:. 

MG01330.1 	HENVS-------------------------------------PKAVQVAVKNSSAGLPLP
NCU03032.1	VEHIDAIATESPRSSPRLVGKHSSSATNALAFAVGRTAPEMRSKAFNTAIENISANLPLP
FG00742.1 	VERIDALVTESPYLYP-------ANHGDALTRAIERSAPEIRSKAFLTAVSNLSSNLPLP
AN5914.1  	IEKLDASFTESPYLSP-------ANHREEMNNAIIQSTQEIHSKAYESAVVNLSANLPLP
          	 *.:.:  :.:.   .       :.  .    :   :: .  .**   *: * *:.****

MG01330.1 	SFFYQNHAWRIYYDDQLRKHKQFNDEYIYAFTWEDTRVDARLLKISSDDVVLAITSAGDN
NCU03032.1	SFFYQNHHWRIYYDDQLPKHTQFNDEYIYAFTWEDSRVDRELLNLGPDDVVLAITSAGDN
FG00742.1 	SFFYQNHHWRIYYDEQLPKHTQFKDEYIYAFTWEDTRVDERILKLGADDKVLAITSAGDN
AN5914.1  	AAFYQNHHYRIFYNDLLPKHTQFKNEYIYAFNWEDPRVDHRLLNIKRDDVILAITSAGDN
          	: ***** :**:*:: * **.**::******.***.*** .:*::  ** :*********

MG01330.1 	ILSYISQSPARVHAVDLNPSQNHLLELKLASYSALDYADFWKIFGEGHHDEFRALLINKL
NCU03032.1	ILSYLMQSPARVHAIDLNPAQNHLLELKVASFTTLDYPDVWKIFGEGKHPDFRSLLISKL
FG00742.1 	ILSYLLQSPARVHAVDLNPTQNHLLELKAASYTALPYGDFWKIFGDGKHPRFRELLITKL
AN5914.1  	ILDYLQKSPRRVHAVDLNPNQNHLIELKVASFIALGHRDVWKIFGEGKHPEFRNLLISRL
          	**.*: :** ****:**** ****:*** **: :* : *.*****:*:*  ** ***.:*

MG01330.1 	SPHLSSRAFQYWFANAHIFT--SKHGLYDTGGSKHAIRAFRWISSAFGVRSAVREMLEAK
NCU03032.1	SPHLSGRAFQYWLSNAHIFTDPAGRGLYDTGGSRYAIRFFRWISTLFFCRSAVRRLLSTP
FG00742.1 	SPHLSGRAFQYWLKNVHVFQNSSGYGLYDTGGSRHAIRVFRWIARIFGLQKAVKQLLQAK
AN5914.1  	SPHLSSQAFQYWLEHSHVFTSSSGRGLYETGGSRHAIKMIRYIFKVFGLEGQVKKLCEAQ
          	*****.:*****: : *:* ..:  ***:****::**: :*:*   *  .  *:.: .: 

MG01330.1 	TLNEQREIWQGRIRPALLSRMVSNFVVSQEQFLWSALGVPKHQLAMLERDHATSKVVS--
NCU03032.1	TLEGQRSIYHTKIRPCLLNRFVNGLVLSSDAFLWSALGVPKNQVAMIEADYHRRSISSST
FG00742.1 	TLNEQREIWRRKIRPALLSKLVCNLVVSQESFLWAALGVPKNQLAMIEADHANSDLVKGP
AN5914.1  	TLAEQRQIWP-RIRAVLLSKPLHWAVVSTEWFAWKAAGVPRNQRNMIVEDYFKRNGLTKD
          	**  **.*:  :**. **.: :   *:* : * * * ***::*  *:  *:   .  .  

MG01330.1 	RGEASN--SRTSAIWQFMSDTLDPVAHDTHIAEENPYYYVTMAGKFSPR~CHPDYLSPAA
NCU03032.1	TPSSKEKPSRAEAILHYTTSTLDPVLSTSHLASDNPYYLVCVLGQYTRQ~CHPDYLSPAA
FG00742.1 	KPAAKN--TRSHAIWHYMVNTLDPVAEETHIAADNPYYYVCMDGKFSPK~CHPDYLSPRA
AN5914.1  	MKNAKD--ISGKSIWEYVVDTLDPVVHETMISNDNYFYYLCLKGQFSKR2CAPAYLSPQA
          	   :.:      :* .:  .*****   : :: :* :* : : *::: : * * **** *

MG01330.1 	HAKLSRRGALEDRIRIHTEEIDVVLAGLAPGSLTVAV~VMDSMDWFDPKE-------PGV
NCU03032.1	HSILSAPGAFDG-LRIHTDEIQEVLARFQPGTLTVAV~VMDSMDWFDPPSPEEEKEGRGK
FG00742.1 	HAKLSRPNALDG-LRIHTDELEEVIARITPGTLTVAV~VMDSMDWFDTG--------SRA
AN5914.1  	HVKLSSPGAFDG-LRIHTDEINEVIGRITPRSLTIAV0IMDSMDWFDPEG--------TE
          	*  **  .*::. :****:*:: *:. : * :**:** :********.            

MG01330.1 	AAAQISKLNRALKPGGRVLLRSAGLKPWYVEEFAKLGFAPKRVGCRS---PETRCIDR~V
NCU03032.1	AREQVRRLNRALKVGGKVLLRSAGVEPWYVRVFVEEGFGARRVGCRESGRGDQECIDR~V
FG00742.1 	AAAQITKLNRALAMGGRVLLRSSALTPWYIKEFEGHGFTPKRHGAR----IDGACIDR~V
AN5914.1  	ATAQAQKFNQALKMDGRILLRSASIEPWYIKNFEENGFSARRVGAR----FSGTCIDR2V
          	*  *  ::*:**  .*::****:.: ***:. *   ** .:* *.*     .  **** *

MG01330.1 	NMYASCWLLTKTGGLPPPTPGMEGEAL0F2VIVNVTTTDSRNLVEKQNVLSKPGAQQTGL
NCU03032.1	NMYASCWILEKMEDLEELVDSA-----~-~------------------------------
FG00742.1 	NMYASCWICTKSENLPPPTPEMDRAGG~C~EITSLTI-----------------------
AN5914.1  	NMYASTWICTKTRELQRPTQDRRSNSV~E~YLEI--------------------------
          	***** *:  *   *   .      .                                  

MG01330.1 	IFNLCQSENIHADGKAMRMSIYASKDV
NCU03032.1	---------------------------
FG00742.1 	---------------------------
AN5914.1  	---------------------------
          	
```
